# Supplementary material for: Population dynamics and species composition of maize field parasitoids attacking aphids in northeastern China
Source: PLoS One. 2020 Dec 17;15(12):e0241530. doi: 10.1371/journal.pone.0241530 (PMC7746195; doi:10.1371/journal.pone.0241530)
Supplement: Attachment — Submitted filename: Response to Reviewers.doc [file pone.0241530.s001.doc]

Dear Editors,

Thanks for your decision, and all of us are grateful for valuable comments from two reviewers. We especially appreciate their responsible spirit, and we will try our best to revise our article according to reviewers and resubmit our paper again. We have made changes in accordance with the PLOS ONE's style requirements of the journal. We thoroughly copyedit our manuscript for language usage, spelling, and grammar.

Data Availability Statement: We have no supporting information files. All relevant data are within the manuscript.

The answers to reviewers’ questions are followed:

Reviewer #1: Thanks for your valuable comments.

1. For your **question 1** “The authors should carefully check the References sections, ensure that the formatting of author names and scientific species names were standardized, for example references 15, 31, 32. Moreover, some reference cited is not reporting on M. miscanthi, reference 20.”

We carefully checked the references in the revised manuscript, ensure that the formatting of author names and scientific species names were standardized

2. For your **question 2**. “What molecular method does the authors use to distinguish the different species.”

We mainly used morphological identification method to identify aphids and their parasitic wasps. One of them could not be identified through morphology, so we identified it combining molecular identification. We use mitochondrial COI as a DNA barcode. Lep F1（5’-3’ATT CAA CCA ATC ATA AAG ATA TTG G）and Lep R1（5’-3’TAA ACT TCT GGA TGT CCA AAA AAT CA）. After the gene sequence comparison, we found that *Lysiphlebus testaceipes* with a gene sequence similarity of 92.55% consisted with the result of morphological identification.

3. For your **question 3.** “Please unify the font format in Table 3.”

We carefully unified the font format in Table 3 in the revised manuscript.

4. For your **question 4**. “In Abstract section, the population dynamics of maize aphids and parasitoid wasps were calculated in two consecutive years, 2018 and 2019, but in Results section, I only find the data for parasitoid wasps in 2019 years, please explain the reason.”

We have already conducted the population dynamic analysis of aphids and parasitic wasps in 2018-2019, but in 2018, the number of parasitic wasps was very few because of weather and sample site selection issues.

5. For your **question 5**. “Why did the authors assess the relationship between aphids and parasitoid wasps, what about other parasitic predators of aphids?”

Because parasitic wasps were limited information exists about their species composition, richness and seasonal dynamics in northern China. This study provides important information for the establishment and promotion of aphid biological control in maize fields. During our two-year research, we also conducted a population dynamic survey of predatory natural enemies. Our article mainly studies the relationship between parasitic natural enemies and aphids in maize fields. Nextly, we will focus on the relationship between predatory natural enemies and aphids based on your suggestions.

Reviewer #2: Thanks for your valuable comments.

1. In accordance with your suggestion, the title revised as “Population dynamics and species composition of maize field parasitoids attacking aphids in Northeastern China.”
2. We revised the sentence “and it is predicted that, by 2050, the demand for maize in the developing world would will double” to “by 2050, the predicted demand for maize in the developing world would be doubled.”
3. “Can add any reference in world or china for losses caused by aphids in Maize?”

Some references have been added based on your suggestions. (Huang W, L K. Comprehensive control technology of *Rhopalosiphum maidis*. Bulletin of Agricultural Science and Technology. 1997;7:31-33. Wang Y, Xu J, Su L. Spatial dynamics of *Rhopalosiphum maidis* population. Journal of Northwest A & F University. 2002;4:55-58. Patil V. P. Population dynamics, yield losses and chemical control of aphid [*Rhopalosiphum maidis*(Fitch) on maize](http://14.139.56.90/handle/1/5810024069). College of Agriculture, Junagadh. 2012;7:107.)

4. We revised “higher population fecundity” to “higher population fecundity rate”.
5. We replaced “suite” to “bundle of”.
6. We added the references for this “However, few have systematically studied the control effect of maize field parasitoids on maize field aphids.”(Ali, Abid, Nicolas Desneux, Yanhui Lu, and Kongming Wu, 2018. Key aphid natural enemies showing positive effects on wheat yield through biocontrol services in northern China. Agriculture, Ecosystems & Environment. 266: 1-9. Abid Ali, Nicolas Desneux, Yanhui Lu, Bing Liu, Kongming Wu, 2016. Characterization of the natural enemy community attacking cotton aphid in the Bt cotton ecosystem in northern China. Scientific Reports.)
7. We removed space between “aphelinids” and “and four species of aphelinids.”
8. We revised “ten different hyperparasitoid species were collected in cotton fields.” to “ten different hyperparasitoid species were collected from cotton fields.”
9. We keep the same trend for all scientific names.
10. For your question, “However, research on parasitoids in maize fields is still scarce. You might mean in northeastern? Or also one factor could be the change in transgenic maize introduction and now different species composition or dynamics or may be due to less area under cotton in northern China?”

Research on parasitoids in maize fields is still scarce in northeastern China.

11. We revised “ their parasitoids in 3 maize fields in 2018 and 2019 in northern China.” to “and their parasitoids in three maize fields during 2018 and 2019 seasons in northeastern China.”
12. “Could you add a map with these locations showing the northeastern locations?”

We had already added a map with these locations.Please see the revised manuscript.

13. “You may follow the paper published by Ali et al 2016/2018 for experimental design or sampling in details. You shall add the five sampling method reference as (Ali et al., 2018, 2016).”

We had already followed the paper published by Ali et al 2016/2018 for experimental design or sampling in details shall. We added detailed for experimental design and sampling in details. Please see the revised manuscript.

14. “Tables could be represented well in graphs rather tables and also tables could work for percentage.”

We have a lot of data, and we can clearly see the occurrence of aphids in each period with the table.

15. We have determined the consistency of objectives, methodology and results sections.
16. Is it Jilin province or norther eastern?

Our experimental fields were selected in the Jilin Province region of Northeast China

17. We had already gave future suggestions in the conclusion section.（Our next focus is to conduct laboratory studies on the parasitic function to clarify the reasons for the low parasitic rate recorded in the field and to continue to improve understanding of the parasitic bee species in the maize field.）

18. We revised “We are much thankful to our partners for their support of sample collection, insect rearing and laboratory assays” to “We are much thankful to our partners for their support in sample collection, insect rearing and laboratory assays.”

19. We checked the references carefully according to the format and spelling mistakes.

Sincerely

Dr. Yuan
